# Trophoblast Fusion in Hypertensive Disorders of Pregnancy and Preeclampsia

**DOI:** 10.3390/ijms26072859

**Published:** 2025-03-21

**Authors:** Ikram Benouda, Daniel Vaiman, Francisco Miralles

**Affiliations:** Institut Cochin, U1016, INSERM, UMR8104 CNRS, Université de Paris, 24 rue du Faubourg Saint-Jacques, 75014 Paris, France; ikram.benouda@inserm.fr (I.B.); francisco.miralles@inserm.fr (F.M.)

**Keywords:** preeclampsia, hypertension, pregnancy, trophoblast

## Abstract

Trophoblast fusion into the multinucleated syncytiotrophoblast (SCT) appears as an inescapable feature of placentation in mammals and other viviparous species. The trophoblast cells underlying the syncytium are considered a reservoir for the restoration of the aging peripheric structure. The transition from trophoblasts to SCTs has to be tightly regulated, and could be altered by genetic anomalies or environmental exposure. The resulting defective placental function could be one of the causes of the major placental diseases, such as preeclampsia (PE) and Intra-Uterine Growth Restriction (IUGR). This review attempts to take stock of the current knowledge about fusion mechanisms and their deregulations.

## 1. The Placental Materno-Fetal Interface

### 1.1. Various Mammalian Placentas

**Figure 1:**
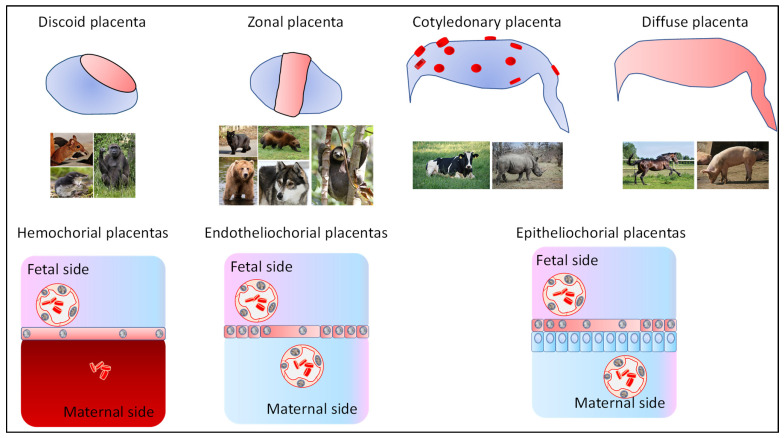
Mammalian placentas appear with different shapes and structures. There is no direct connection between placental type and evolutionary proximity. For instance, Rodents, Insectivora, and Primates share the same hemochorial placental structure despite more than ~80 million years since their last common ancestors. The epithelial maternal cells in epitheliochorial placentas are shown in blue, and the trophoblast cells (that fuse locally, or totally, in hemochorial placentas) are shown in pink.

The placenta is a temporary organ that is essential for the proper progression of pregnancy. It facilitates the exchange of nutrients and oxygen between maternal and fetal blood. Present in Metatherians (Marsupials) and Eutherians, as well as in some non-mammalian species where the offspring develops inside the maternal organism, the placenta varies in appearance. In mammals, placentas can have different shapes, including discoid, diffuse, multi-cotyledonary, and zonal forms (Figure 1).

The number of cell layers separating maternal and fetal blood can range from six layers in epitheliochorial placentas, as found in equids, to just three layers in hemochorial placentas, such as those in humans.

In addition to its primary function of reducing the mother’s immune response to the semi-allogenic fetus ([1,2]), the placenta serves as an important protective barrier. It protects the fetus from certain toxic substances and infectious agents, including viruses and bacteria. Furthermore, the placenta produces hormones such as chorionic gonadotropin (hCG), placental lactogen (hPL), estrogens, and progesterone. These hormones are crucial for maintaining pregnancy and preparing the mother’s body for childbirth and breastfeeding.

Most of these functions occur in a specific structure of the placenta known as the syncytiotrophoblast (SCT). The SCT is a syncytium formed by approximately 10 billion nuclei in mature human placentas, all sharing the same cytoplasm. In the mature human placenta, the SCT comes into direct contact with maternal blood, making it the primary surface for maternal–fetal exchange.

### 1.2. Placental Development

Shortly after its attachment (8 days post coitum in humans), the blastocyst (composed in this stage of two major cell types (the trophectoderm and the Inner Cell Mass) begins to invade the decidua by proliferation and differentiation of the polar trophectoderm (the part of the trophectoderm that is nearby the Inner Cell Mass), by opposition to the mural trophectoderm on the opposite pole of the blastocyst. Two primitive trophoblast lineages arise from the trophectoderm: the primitive cytotrophoblast (CTB) and the primitive SCT. The primitive SCT results from the fusion of the underlying CTBs and forms an expanding multinucleated mass that contributes to invasion of the decidua through the de novo expression of matrix metalloproteases, such as Matrix Metalloproteinase 9 (MMP9) [3].

Later on (~12 dpc), the primitive CTBs proliferate and project into the overlying primitive SCT, forming the primary villi. The CTBs continue to proliferate from the tips of this initial villi. Then (around 28 dpc), the basic villous structure of the placenta has formed, consisting of a mesenchymal core containing fetal blood vessels, placental macrophages, and connective tissue, surrounded by a specialized epithelial bilayer composed of inner CTBs and an outer SCT [4]. This mature SCT differs from the primitive SCT since it is not invasive but rather acts as the interface between the maternal circulation and the placenta. As mentioned, the placenta has an endocrine function and the SCT is the major part of this endocrine organ, secreting hormones and diverse proteins. This syncytium is highly polarized, and the apical membrane is covered with microvilli which increase considerably the surface of contact with the maternal blood. Moreover, the microvilli are enriched in receptors for growth factors and hormones. Both the apical and basal membranes are packed with transporter proteins. The basal membrane is in contact with the CTB layer beneath. However, the SCT is terminally differentiated and fragments of the SCT are continuously shed into the maternal circulation. The regeneration of the syncytium along pregnancy is ensured by the underlying CTBs through a cell fusion process.

### 1.3. Syncytialization Is Systematically Present in the Various Placentas Throughout Evolution

While viviparity is prominent in mammals, this mode of reproduction occurs throughout the evolutionary tree of vertebrates (as well as outside vertebrates). Forming a placenta exists for ~2% of Reptiles [5] (with several lizard species such as Mabuya [6]), as well as several fish species, especially several sharks such as Rhizoprionodon taylori [7]).

Understanding all the mechanisms of trophoblast fusion cannot be achieved without mentioning the seminal series of works published from 2003 by Thierry Heidmann’s team, emphasizing the discovery of a syncytin gene in several placental mammals (Primates [8], Muridae Rodents [9], Carnivores [10], Ruminants [11], Insectivores [12], but also in Metatherian Marsupials [13], and even outside mammals in the Mabuya lizard [6]). All these staggering works revealed that during the biological history of every one of these species, at a certain moment, an external endoretrovirus nested inside the host genome. Then, the selection advantage that was brought was such that, in every case, the virus settled and was maintained, suggesting that this advantage was indeed significant in terms of fitness for the recipient species.

A major question, in evolutionary terms, is therefore to understand the actual advantages provided by the syncytialization of the trophoblast. Syncytialization is systematic in humans, but is merely partial in numerous other species, such as ruminants, like cattle, where syncytial plaques generally cover small areas within placentomes. Nevertheless, whatever the extent of the syncytium, it appears as an inescapable feature of placentas. A more sophisticated and recent study suggests that macropinocytosis could be specifically allowed by a syncytialized layer of cells [14]. This mechanism enables the transfer of fluid-phase-derived molecules from one side to the other side of the cell/syncytium membrane, especially large molecules (>70 kDA) [15], and is partly under the control of the mTOR (mammalian Target Of Rapamycin) signalization cascade [16]. In the latter paper, the authors show that amino-acid shortage (AAS) triggers improved syncytialization, and this was also connected with a decreased mTOR signaling, synergized between syncytialization combined with AAS. Rapamycin treatment simultaneously enhanced trophoblast fusion and macropinocytosis. Reciprocally, the induction of mTOR signaling with MHY1485 inhibited the effect of AAS, thus reducing syncytialization. It is therefore possible that the systematic selection of fusion mechanisms in the placenta is a requisite to make possible the efficient transfer of specific nutrients (large molecules) through the barrier. In vivo, mice were treated by intraperitoneal injection of Rapamycin, which reduced P-S6K signaling (as in cell models). When pregnant mice were treated, the injection led to a decreased fetal and placental weight, and an increased expression of Glial Cells Missing-1 (Gcm1, a major trophoblast-specific transcription factor) and Syncytin A and Syncytin B (Syna and Synb). EIPA (a macropinocytosis inhibitor) was also used in mice and it was possible to see that placenta and fetal weights were reduced by this inhibitor, aggravating the effects of Rapamycin treatment. This paper suggests that syncytialized trophoblasts are key for making micropinocytosis effective, compensating for defects of nutrients when the amino acids are rare, which might be the case at the end of gestation, where the requests may mimic maternal malnutrition conditions. In the rat model, IUGR (Intra-Uterine Growth Restriction) induced by a low-protein diet indeed induced mTOR expression 2.16-fold [17], strengthening the idea of an adaptive mechanism to compensate the AAS. A 2010 review indeed evoked the potential of mTOR signaling as essential components of trophoblast function [18]. There are examples in the literature of attempts to leverage mTOR signaling as a therapeutic approach in the context of preeclampsia/fetal growth restriction. For instance, Tanaka and coworkers utilized the Tadalafil PDE5 inhibitor to modulate TOR signaling, thus improving fetal growth in the context of preeclampsia induced by L-NAME treatment [19,20]. Similar attempts to positively alter the mTOR cascade were partly successful using pravastatin treatment, which allow alterations of the p-mTOR/mTOR ratio.

### 1.4. How Do We Study the Syncytialization Process?

**Figure 2:**
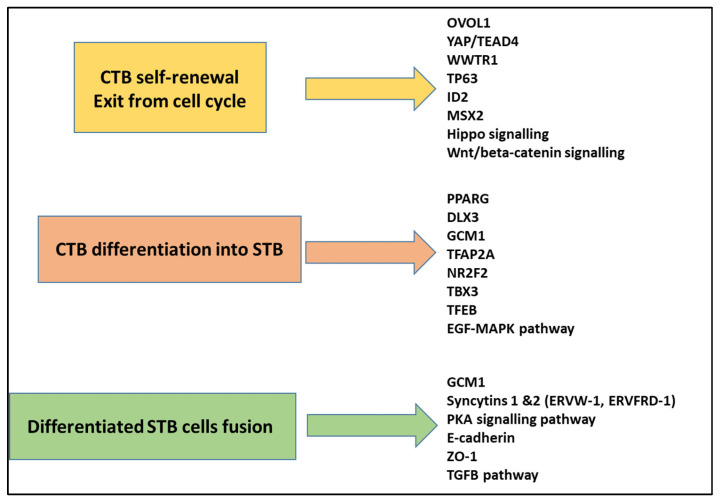
An overview of the major genes implicated in the fusion process classified into the three steps leading to trophoblast fusion. Details concerning the role of each of these genes/pathways are given in Section 1.4.

The CTBs beneath the SCT are considered “stem cells” because they are proliferative and can differentiate into SCTs or EVTs. The SCT is regenerated throughout gestation by the constant fusion of these CTBs. This process involves at least three steps: the CTBs must exit the cell cycle, undergo differentiation, and express fusogenic factors (Figure 2). What signals initiate this process and how it is orchestrated have been and still remain a matter of intense research, certainly in part because the numerous genes involved in the process are not known. Our comprehension of the mechanism of human CTB fusion has largely relied on the use of different models [21]. These include immortalized cell lines derived from choriocarcinomas (BeWo that can be induced to fuse through AMPc cascade activation, JAR, and JEG-3) and others [22]. Like the CTB in the actual placenta, they fuse into SCT-like cells, producing placental hormones (hGC, placental lactogen, or others). Other cell lines, such as JEG-3 or JAR, respond in terms of the transcriptome or proteome in response to FSK, but do not fuse [23,24]. Also, the advent of transcriptomic technologies (DNA-microarray and RNA-seq analysis), as well as other omics technologies (epigenomics, proteomics, and metabolomics), is playing a pivotal role in obtaining a global view of the mechanisms involved in the formation of the SCT [25]. Thus, single-cell/single-nucleus RNA-sequencing (sc/snRNA-seq) analyses of the placenta has been used to identify cell-type-specific gene signatures. Differentiation trajectory analysis based on sc/snRNA-seq data has allowed the identification of cell subtypes and transcriptional changes involved in the transition of CTBs towards SCTs [26,27,28,29]. To note, as well, the extracellular matrix may influence the fusion capability of trophoblasts [30].

In the BeWo model, knockout/knockdown experiments demonstrated the implication of genes involved in the fusion defects, such as *TMEM16F* [31], *ATP11A* [32], *ANXA1/ANXA5* [33,34], *HTRA4* [35], *ZO1* [36], *PAR6* [37], *CX43* [38], cadherin/catenin themselves and their regulators, *TWIST* [39], *ECAD* [40], *Calreticulin* [41], *ADAM12* [42], and *GAL1* [43,44].

Indeed, unpublished results from our own lab from RNA-seq of single nuclei revealed a subpopulation of cells in the process of fusing from cytotrophoblasts into syncytiotrophoblasts, characterized by a set of 436 specific genes. The systematic downregulation/KO of these will reveal how they actually influence fusion, in a more agnostic way than what was previously performed. This could pave the way toward comprehensively building the cascade of cellular events leading to fusion in trophoblasts.

To acquire the competence to fuse, CTBs must first exit the mitotic cell cycle [45]. Therefore, they must repress genes involved in the maintenance of the progenitor state such as *TEAD4*, *YAP*, *TP63*, *GATA3*, *ID2,* and *MSX2*. At present, the signals initiating the exit of the CTBs from the mitotic cell cycle are not known. OVOL1 has been identified as a key regulator of SCT development [46]. This transcription factor represses the expression of genes required to maintain the CTBs in a proliferation state such as *MYC*, *ID1,* and *TP63*. Also, the transcriptional activator complex YAP-TEAD4 of the Hippo pathway induces the expression of genes promoting *CTB* self-renewal. On the other hand, this complex can also bind to the histone methyltransferase *EZH2* and repress the expression of SCT-specific genes [47]. Another component of the Hippo signaling, WWTR1, participates in CTB self-renewal through the direct regulation of TP63 expression [48]. Also, the knockdown of WWTR1 results in the repression of several members of the Wnt/beta-catenin signaling (WNT3, WNT4, WNT5B, WNT7A, WNT8B, WNT9A) and results in the loss of proliferation and self-renewal capacity of the CTBs [48]. Similarly, the knockdown of MSX2 leads to a loss of proliferation and self-renewal of the CTBs and activates spontaneous SCT differentiation and syncytialization [49]. Another promoter of cell cycle arrest is the p57/Kip2 in the G1/G0 phase, with the gene being negatively regulated when the cells fuse [50]. BCL6, involved in cell division, also influences trophoblast fusion [51]. The general function of this factor in cell proliferation does not render it a pivotal factor of the ultimate trophoblast differentiation in SCTs.

Following cell-cycle arrest, the CTBs initiate SCT differentiation. Several transcription factors and epigenetic regulators are known to be involved in this process, including *PPARG*, *DLX3 GCM1*, and *TFAP2A.* The blockade of the activity of *PPARG* (Peroxisome Proliferator-Activated Receptor, gamma) downstream of the EGF-MAPK (Epidermal Growth Factor–MAP kinase) pathway in BeWo cells results in impaired SCT differentiation [52]. DLX3 (Distal-less Homolog, 3) is required for placentation, and it is involved in the secretion of chorionic gonadotrophin (hCG) [53]. This transcription factor (TF) binds to the promoter and regulates the expression of several markers of the SCT such as CSH1, HSD3B1, and PGF [54]. The transcription factor activator protein-2 alpha (TFAP2A) has been identified as a critical regulator of biochemical SCT differentiation. The expression of a dominant-negative version of TFAP2A significantly inhibited the induction of essential SCT markers, including hCG, the PSG family of genes, PGF, and CYP11A1 [55]. Interestingly, the inhibition of TFAP2A signaling did not affect cell fusion, demonstrating the uncoupling of the biochemical and morphological stages of SCT differentiation. In addition, it is known that *TFAP2A* is regulated by NR2F2. The induction of NR2F2 promotes the expression of TFAP2A, and conversely, NR2F2 depletion reduces it, resulting in impaired SCT differentiation [56]. TBX3, a member of the T-box transcription factor family, is activated during the middle stages of human pluripotent stem cell differentiation into trophoblast ectoderm. An scRNA-seq analysis combined with loss-of-function experiments in the JEG-3 cell line demonstrated that TBX3 is required for both syncytium formation and biochemical differentiation as it regulates the expression of hCGA and hCGB, Syncytin-1 (*ERVW*-1), and other HERV-derived genes (*ERVV*-1 and *ERVV*-2) [57]. Recently, TFEB (transcription factor EB), a bHLH-leucine zipper transcription factor belonging to the MiT/TFE, has been identified as essential for both differentiation and syncytialization [58]. In vivo analyses revealed that homozygous TFEB KO mice display reduced SynTII formation. In vitro analysis using TFEB BeWo knockout cells stimulated with FSK shows severely impaired syncytialization, a reduced expression of genes ERVW-1 and ERVFRD-1 encoding the fusogenic proteins Syncytin-1 and Syncytin-2, and enzymes involved in steroidogenic pathways, such as CYP19A1.

Another requisite for the syncytialization process is the expression of fusogenic proteins. The use of immortalized cell lines (BeWo cells) made it possible to identify a crucial event occurring during SCT formation. Stimulation by FSK generates an increase in cAMP, leading to the activation of the protein kinase A (PKA) signaling pathway, which, in turn, promotes the expression of Glial Cells Missing-1 (GCM1) [59,60]. GCM1 directly drives the expression of the genes encoding the fusogenic proteins syncytin-1 (ERVW-1) and syncytin-2 (ERVFRD-1). Syncytin-1 binds to its receptors ASCT1 and ASCT2 expressed by the CTBs. Syncytin-2 binds to MFSD2 expressed by the SCT. In the mature placenta, the SCT produces hCG, which binds to the LH-CG receptor expressed by CTB, which results in the production of AMPc and the activation of the PKA signaling pathway which will upregulate GCM1 [61]. Thus, the SCT itself can act as a positive regulator of syncytialization. On the other hand, the SCT can inhibit the syncytialization process through the production of TGFβ [62]. The activation of PKA signaling through hCG is also coupled to the phosphorylation of Connexin 43 (Cx43) through Ezrin, which induces the opening of gap junctions and the transfer of fusogenic signals between the CTBs and the SCT [38,63]. The syncytialization process also involves important modifications in the cytoskeleton of the differentiated SCT. Thus, E-cadherin mediates CTB aggregation, while cadherin-11 is required for syncytialization [64,65]. The alterations of the membrane during the process strongly suggest that membrane component equilibrium is essential in placental function, especially cholesterol (as exhaustively put in perspective in a recent review [66]); transporters of this molecule (ABCA1, ABCG1) are indeed deregulated during the fusion process [67].

The number of factors involved in placental syncytialization is in fact large, since this is a quite complex biological mechanism. In fact, fusing cells will need membrane modifications, in cell signaling following cell cycle exit and associated with specific differentiation cascades, as nicely reviewed recently [68]. It should be noted, as well, that ERVV-1, encoding Syncytin1, has recently been shown to present functions that are relevant to placental health, but disconnected from fusion mechanisms.

### 1.5. SCT Heterogeneity and Life Cycle

The SCT undergoes a highly regulated turnover as aging or damaged nuclei are replaced by the underlying CTBs through the process of cell fusion. Therefore, the syncytium contains nuclei with different levels of transcriptional activity which may represent different stages of maturity. Recently, new studies using single-nucleus RNA sequencing (snRNA-seq) and single-nucleus Assay for Transposase-Accessible Chromatin-sequencing (snATAC-seq) technologies have started to reveal the dynamic heterogeneity of the SCT nuclei, their differentiation route, and potential roles [69]. This study suggests the coexistence of different SCT nuclei with possible distinct biological functions inside the same cytoplasm. Thus, the analysis of early (6–9 weeks) and late (38–39 weeks) gestation placentas has revealed the existence in both stages of SCT nucleus subtypes displaying specific transcriptional signatures. In early placentas, they identified six SCT subtypes, among which one cluster expressed high levels of PAPPA and another one expressed high levels of FLT1. In late placentas, this heterogeneity was consistently conserved; however, PAPPA was increased in most SCT subtypes, while the expression of FLT1 was considerably downregulated, and only a small fraction of SCTs maintained FLT1 expression. Further studies will allow the investigation of if/how this SCT heterogeneity is impacted in the placental pathologies, such as preeclampsia. Nevertheless, the nuclei in the SCT ultimately go through a senescence process, leading to their shedding into the maternal circulation as syncytial knots and sprouts [70]. Interestingly, SCT apoptosis and autophagy-induced cell death have been found increased in both preeclampsia and IUGR ([71]). Also, consistently with these observations, circulating syncytial debris appears to be increased almost twofold in women with preeclampsia ([72,73]). A recent snRNA-seq analysis of human placentas issued from normal and preeclamptic pregnancies has identified different transcriptomic states of the SCT nuclei associated with the expression of secretory senescence factors ([74]). Moreover, the authors found that the circulating levels of these factors can predict early-onset preeclampsia (eoPE) before clinical symptoms and diagnosis. These suggest that premature SCT senescence could be a major factor in the development of eoPE.

## 2. Trophoblast Fusion in Diseases of Placental Origin

In addition to the aforementioned link between Intra-Uterine Growth Restriction and potential placental macropinocytosis defects, placental fusion defects may also contribute to preeclampsia, which in turn causes IUGR in approximately 30% of cases.

Among the various causes of preeclampsia, placental aging has been suggested as a possible cause of the disease; a possible fundamental difference between late-onset and early-onset preeclampsia has been strongly advocated by the late Chris Redman in recent reports [65], but this hides a much richer complexity. They suggest that a specific syncytiotrophoblast stress is at the center of the pathogenesis of the disease (these stresses include, in isolation but more likely in combination, oxidative stress, ER stress, mitochondrial stress, apoptosis, autophagy, syncytiotrophoblast rupture, complement deposition, and necrosis). Other stresses could be added, such as replication stress, which occurs when cells divide too quickly or too slowly. A major difference reported by the authors is the idea that in early-onset preeclampsia (EOPET), oxidative stress is a starting point for all these stresses, whereas in late-onset preeclampsia (LOPET), placental hypoxia is more at work in generating the stresses common to the two major clinical forms of PE. Stresses could also be associated with viral infection (itself connected to interferon cascade activation). IFITM molecules (IFN-induced transmembrane proteins (IFITMs)) [66]), which inhibit viral entry steps, are also able to inhibit syncytin-mediated fusion, also modulating trophoblast invasion [67].

Certain aspects connecting trophoblast fusion (including apoptosis, epigenetics, hypoxia, alterations of the ligand/receptor interaction between trophoblasts prior to fusion) and pathology are presented below.

### 2.1. Hypoxia and Fusion

From the implantation until the first trimester of pregnancy, the development of the embryo and early placenta occurs under low oxygen tension (~2–3% O_2_). During this period, early EVTs differentiate, migrate, and invade into the spiral arteries to form plugs that prevent the maternal blood from flowing into the intervillous space, thus maintaining a low-oxygen environment. By the end of the first trimester, these plugs are removed and endovascular trophoblasts (eEVTs) begin migrating proximally along the vessel to cause spiral artery transformation into “low-resistance” vessels, an important process to establish a high flow of maternal blood circulation, and therefore rapidly increasing the oxygen levels (~8 O_2_) in order to support fetal growth. It is now well established that hypoxia plays a crucial role during early placental development. There is still some controversy regarding the effects of hypoxia on the different placental lineages. However, several studies have demonstrated the importance of a hypoxic environment for the blastocyst to attach to the uterus, initiate placental proliferation through the invasive trophoblast cells, and maintain the stem cell state of trophoblasts, reviewed in Burton and collaborators [70]. Thus, a major role of hypoxia during early placentation would be to ensure the necessary expansion of the pool of trophoblast stem cells. A recent study has shown that in human trophoblast stem cells (hTSCs), the transcription factor GCM1 is downregulated by hypoxic conditions, which seems to maintain hTSC proliferation while preventing differentiation into both SCTs and EVTs [75].

Hypoxia/reoxygenation injury is a hallmark of preeclampsia. Thus, placental hypoperfusion is known to cause increased placental oxidative stress and nitrosylation of proteins, leading to syncytium damage, an impaired balance of angiogenic and anti-angiogenic factors, and the release of pro-inflammatory molecules into the maternal circulation. It has been shown that a culture of primary CTBs and multiple CTB cell lines under low oxygen inhibits fusion and the expression of specific SCT markers [76,77]. Recent studies point out that hypoxia could directly impair the formation of the syncytium. Thus, the analysis of pathological placentas has revealed an increased number of Hypoxia-Inducible Factor 2A (HIF2A)-positive nuclei in the syncytium compared to normal human placentas. The specific involvement of HIF2A was confirmed in primary human cytotrophoblasts rendered deficient for Hypoxia-Inducible Factor 1A (HIF1A) or HIF2A. Silencing HIF2A increased the expression of main syncytialization markers as well as differentiation and syncytium formation. On the contrary, HIF1A silencing did not alter the syncytialization process. Moreover, in BeWo cells, the induction of HIF2A expression repressed forskolin-induced syncytialization [78]. The mechanisms involved in altered syncytialization through poor oxygenation remain unknown, but recent studies have started to shed some light. The expression of suppressyn, a human endogenous retroviral protein that inhibits the fusogenic activities of syncytin-1, is controlled by DNA-methylation and oxygen concentration through the transcription factors HIF1A and HIF2A. Using the BeWo and JEG3 cell lines, it has been shown that the HIF1A and HIF2A proteins bind to the suppressyn promoter sequence and are involved in its increased expression in response to low oxygen levels [79]. A genome-wide analysis of ARNT/HIF targets has revealed a total of 562 genes potentially implicated in impaired human SCT formation under hypoxia. Among these are ERVH48-1 encoding suppressyn (mentioned above) and BHLHE40. Further studies are expected to elucidate how an altered expression of these genes under low oxygen can affect SCT formation [80]. The placental-specific serine protease HTRA4 is involved in protein quality control and syncytialization [35], and appears to be upregulated in preeclampsia, particularly EOP [81]. In EOP, the expression of HTRA4 positively correlates with endoplasmic reticulum stress [82]. Under normoxia, HTRA4 overexpression does not alter ERS levels in BeWo cells; however, hypoxia/reoxygenation or treatment with an ERS inductor increases HTRA4 expression. Under these conditions, the upregulation of HTRA4 suppresses the levels of syncytin-2 and bHCG in the presence of FSK, and this was exacerbated after ERS. The authors of this study conclude that ERS enables syncytialization by upregulating HTRA4 but that the excessive expression of HTRA4 (as induced by hypoxia/reoxygenation) and preexisting ERS impairs syncytialization [82].

The emergent role of oxygen tension in the control of the self-renewal of the hTSC and their differentiation in either EVTs or SCTs opens new horizons to explore placental pathologies such as preeclampsia where maternal vascular malperfusion is known to play a major role.

### 2.2. Apoptosis and Fusion

Apoptosis, programmed cell death, is known to play important roles in organ development, and this includes the placenta and trophoblast differentiation. A subcategory of apoptosis is necroptosis, which presents features that are common to autophagy and necrosis.

Increased ceramide concentrations in the plasma of patients have been evidenced by the team of I. Caniggia [83]. The origin of this could be by an increased activity of lysosomal hydrolase triggered by oxidative stress, eventually featuring sphingolipid storage disorder in the context of preeclampsia. Further work indicated that this increased ceramide concentration leads to an activation of a necrosome complex composed of RIP3, RIP1, and FADD, which inhibits the maturation of Procaspase-8 into Caspase-8. This leads to Mixed Lineage Kinase Domain-Like (MLKL) phosphorylation (with MLKL being a major player of necroptosis), eventually promoting necroptosis. This leads to the premature death of trophoblast cells, thus preventing their normal fusion in SCTs [84].

Amongst the enzyme involved in apoptosis, caspases play a prominent role; in the context of placental development, caspases are involved in tissue remodeling, but also trophoblast fusion and differentiation [85], and therefore in placental diseases such as preeclampsia [86]. Caspases-1, 3, 8, and 10 are increased in PE placentas [87]. The p53 cascade, a major regulator of apoptosis genes (including caspases-3 and 8) is also altered in preeclampsia, and could promote excessive apoptosis (and autophagy) in the context of placental disease. Another recent factor, JAM3 (Junctional Adhesion Molecule 3), has been reported in this respect [88]. The knockdown of this gene increases syncytium formation, and was decreased in the plasmas and placentas of patients affected with EOPET. Thus, proteins at the interface of trophoblasts, as membrane and cell junction elements, are also directly involved in fusion mechanisms. Syncytin-1, when reduced, could also promote apoptosis [89], in the frame of activity of this protein that is disconnected from fusogenic functions.

### 2.3. Epigenetic Regulation of Fusion in the Context of Placental Disease

**Figure 3:**
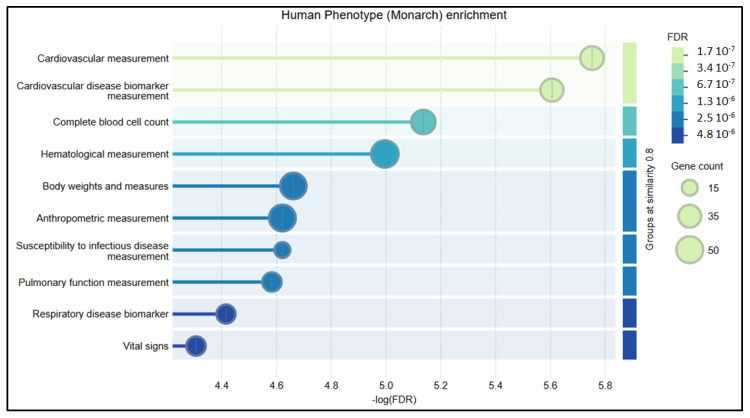
Human phenotypes associated with abnormally methylated genes in the context of preeclampsia are clustered around specific functions.

Placental disease induces the deregulation of numerous placental genes, as has been largely studied in the case of preeclampsia [90,91], with numerous levels of expression regulation, in addition to variants associated with these variations in gene expression, which cover maternal predispositions, placental dysfunctions, and impaired immune tolerance. The regulation of gene expression is under the control of epigenetic modifications. DNA methylation is one of the most stable and best-characterized epigenetic modifications. DNA methylation occurs at the fifth carbon position at a cytosine residue within a CpG dinucleotide, forming 5-methylcytosine. Alterations of methylation compared to a reference state are potentially connected to environmental exposures such as toxic metals, air pollution, chemical compounds, and tobacco [92,93,94,95], and potentially to blood pressure increases during pregnancy [96]. In parallel, DNA methylation plays a critical role in embryonic development. The alteration of DNA methylation patterns has been linked to several diseases. Numerous studies have shown abnormalities in DNA methylation in PE. Some early studies have demonstrated that the global DNA methylation levels were elevated in women with PE compared to normotensive ones [97]. Changes at the gene level have been pinpointed to genes involved in inflammation, placental development [98,99,100], metabolism [101,102], lipid metabolism [103], and immune regulation [104]. Since these epigenetic changes did largely influence placental function and trophoblast behavior, the authors suggest that understanding these changes may contribute to the identification of new biomarkers for an early prediction of PE, and provide insights into therapeutic targets for managing PE. In addition, DNA Methyl Transferase (DNMT) overexpression tends to inhibit trophoblast fusion, while methylation inhibitors present the opposite effect, with the expression alteration of 104 genes, including Syncytin 1, hCG, and the phosphorylated version of CREB, which promotes proliferation and invasion [105]. A re-analysis of these 104 genes performed in the context of this review (Figure 3) demonstrated that the genes are enriched in the several KEGG pathways relevant to preeclampsia (TNF signaling pathway, cortisol synthesis and secretion, and AMPK signaling). In terms of human phenotype, there was an interesting enrichment in cardiovascular diseases, blood cell count, and body weights and measures, with all these cascades being potentially related to the preeclamptic phenotype.

In addition to methylation alterations, some long non-coding RNA and miRNA are important in fusion and altered in preeclampsia, with one of the most documented being UCA1 (Urothelial Cancer-Associated 1) [106]. It has recently been shown that UCA1 originates from the HERVH endoretrovirus [107]. The overexpression of UCA1 impedes human trophoblast syncytialization and is detectable in EOPET placenta [106,107]. It should be noted that the STOX1 transcription factor, the two isoforms of which are STOX1A and STOX1B, oppositely affects trophoblast fusion [34] and alters UCA1 expression. One specific mutation of STOX1 involved in HELLP syndrome (an aggravation of preeclampsia), T188N, specifically affects the expression of UCA1, inducing an increased overexpression 2.91-fold, over the WT version of the gene, strengthening the connections between this lncRNA, trophoblast fusion, and placental disease.

In terms of histone post-translational modifications, epigenome dynamics, particularly cytotrophoblasts from severe preeclamptic patients, present increased H3K27 acetylation [108]. An interesting observation is that, in some cases of severe preeclampsia, the peaks are typical from normotensive second-trimester profiles [108]. This illustrates the fact that an alteration of the chronology of the differentiation along the pathway leading from villous trophoblasts to syncytiotrophoblasts is sometimes associated with aging uchronies. A recent study indicates that specific epigenetic marks (H3K4me3 and H3K9ac) are opposed on trophoblast DNA via galecitn2, known to be reduced in preeclampsia, which is associated with increased fusion in the BeWo cell model [109].

### 2.4. Direct Effects of Fusion

A study was conducted to investigate abnormalities in the expression of Syncytin-2 and its receptor, ASCT2, in the context of preeclampsia (PE) [110]. The research included 60 women diagnosed with PE and 58 pregnant women undergoing pregnancy termination, experiencing preterm birth, or having normal-term deliveries. Using real-time qPCR, they measured the mRNA expression of syncytin 2 and its receptor ASCT2 (also known as SLC1A5, standing for Solute Carrier Family 1 Member 5) in the PE group and the controls. The analysis revealed a significant reduction in syncytin2 mRNA levels in women with PE. Conversely, there was no statistically significant difference in the expression levels of ASCT2 mRNA between the two groups. These observations suggest a specific alteration in syncytin 2 regulation in PE [110]. The authors then concluded that the reduced expression of syncytin but not ASCT2 may be associated with preeclampsia; this was also sustained by the in vitro observation that hypoxia decreases Syncytin but not ASCT2 in vitro. Less obvious genes are also connected to disease; for instance, THBS1 (Thrombospondin1) limits cAMP signaling and, therefore, reduces the fusion in BeWo cells, through a CD36 (thrombospondin receptor)-dependent mechanism [111].

In EOP, the expression of HTRA4 positively correlates with endoplasmic reticulum stress [81,82]. Under normoxia, HTRA4 overexpression does not alter ERS levels in BeWo cells; however, hypoxia/reoxygenation or treatment with an ERS inductor increases HTRA4 expression. Under these conditions, the upregulation of HTRA4 suppresses the levels of syncytin-2 and bHCG in the presence of FSK, and this was exacerbated after ERS. The authors of this study conclude that ERS enables syncytialization by upregulating HTRA4, but that the excessive expression of HTRA4 (as induced by hypoxia/reoxygenation) and preexisting ERS impairs syncytialization [82].

The emergent role of oxygen tension in the control of the self-renewal of the hTSC and their differentiation in either EVTs or SCTs opens a new pathway to exploring placental pathologies such as preeclampsia where maternal vascular malperfusion is known to play a major role.

## 3. Estimating the Importance of the Balance of Trophoblast Renewal/Fusion in Placental Diseases Is a Challenge

**Figure 4:**
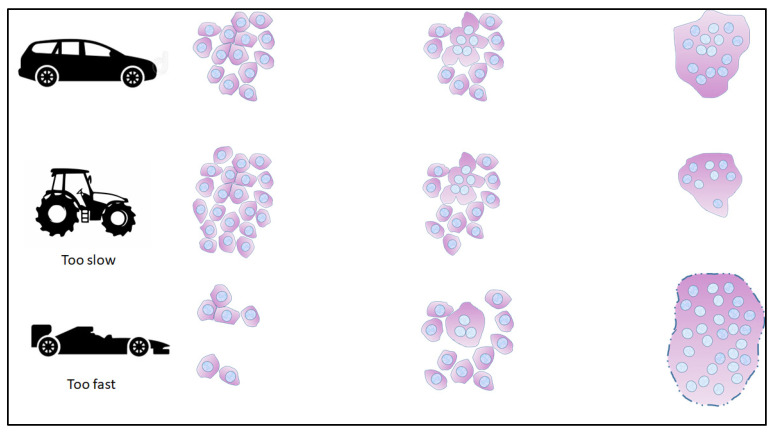
The potential implication of anomalies in the fusion process are represented. The upper lane represents the normal cadency of the process, which potentially helps in adjusting the lifespan of the placenta. The syncytiotrophoblast represented in the last column is still healthy, shedding parts but still possessing some trophoblasts to renew it. In the middle lane, the non-fused trophoblast cells accumulate and lead to the defect in the extension of the formation of the SCT, with a decreased capability for exchange, hormonal synthesis, etc. In the last lane, the process is too rapid, the number of remaining trophoblasts is exhausted, and the large syncytiotrophoblast is senescent too early due to excess membrane shedding. Potentially, both abnormal situations could lead to placental dysfunction and disease.

Trophoblast fusion is the canonical pathway for the differentiation of placental cells, making it possible to generate a functional placenta. The equilibrium between cytotrophoblast renewal and their fusion must be finely tuned to ensure an adequate gestation duration (Figure 4).

It is logical to surmise that this complex mechanism, based on intertwined cellular physiological processes, may be vulnerable to gene dysfunctions, whether triggered by environmental perturbations or genetic variations. However, this does not imply that all cases of preeclampsia are caused by alterations in this renewal/differentiation equilibrium, and estimating the percentage of cases influenced by this factor is a challenging issue.

Among the major factors in aging regulation, SIRT genes play a crucial role and have been shown to modulate aging effectively. Sirtuins 1–7 are deacetylases involved in metabolism and are essential for survival in various organisms, including worms, flies, and mice [112]. For instance, the invalidation of Sirt6 leads to a shortened lifespan in mice, primarily due to an increased NF-kappa B signaling cascade, which results in excessive inflammation and potentially premature aging through H3K9 deacetylation [113].

In the context of the syncytiotrophoblast, sirtuins 1 and 2 are expressed throughout gestation, while SIRT1 is significantly downregulated in preeclamptic placentas affected by premature aging [114]. The differences are specifically observed in the syncytiotrophoblast. SIRT1 protein levels in the placenta were correlated with circulating sFLT-1. Furthermore, the downregulation of this protein in trophoblast cell lines altered their migration and invasion capabilities, while overexpression produced opposite effects [115].

The potential impact of aging on cell equilibrium and the duration of the placenta may have significant implications for the risk of preeclampsia [116]. In some of our unpublished results, we studied the global methylation of placentas using EPIC arrays in preeclamptic cases. Among the samples analyzed, we identified one case of discrepancy between the actual calendar age of the placenta and the age estimated from specific methylated CpG sites associated with aging. Although the sample size is small, this finding suggests that fewer than 20% of preeclampsia cases may be linked to abnormal aging.

A similar study indicated that methylation patterns are associated with early-onset preeclampsia, leading to an apparent gestational age that is two weeks earlier than that calculated from DNA methylation [117]. Given the heterogeneity of the disease, understanding these factors is essential, as they may be relevant to the placental pathology associated with the deregulation of normal senescence pathways.

## 4. Conclusions

Trophoblast fusion appears as pivotal in placental physiology, potentially to allow the specific transfer of nutrients such as through macropinocytosis. It is logical to assume that defects in the fusion process, especially its bona fide rhythm, will alter normal placental function and lifespan, and could sometimes logically result in major placental diseases that are PE and IUGR. To be able to evaluate correctly the part of fusion in the onset of placental diseases, elucidating the complete cascade of the process is compulsory. For this, a first step is the identification of all the genes that are involved. Presumably, these will often be located at the cell membrane, and may be part of various junctional complexes. Re-analysis of GWAS focusing on the screen of the genes specific to trophoblast fusion could be an interesting approach to identify such candidate genes, since it would allow the requested threshold for significance to be decreased due to a limitation in the number of tests to be performed and thus the harshness of corrections for multiple testing. Once such genes are identified, inactivating them in BeWo cell models or in primary trophoblasts either by knockdown with siRNA or knockout by CrispR-cas9 approaches could enrich the panel of genes intertwined in the process of trophoblast fusion in mammals, whose mutations are involved in human placental diseases.
